# Treatment of Fibromyalgia Syndrome: Recommendations of Recent Evidence-Based Interdisciplinary Guidelines with Special Emphasis on Complementary and Alternative Therapies

**DOI:** 10.1155/2013/485272

**Published:** 2013-11-21

**Authors:** Jacob Ablin, Mary-Ann Fitzcharles, Dan Buskila, Yoram Shir, Claudia Sommer, Winfried Häuser

**Affiliations:** ^1^Department of Rheumatology, Tel Aviv Sourasky Medical Center, 64239 Tel Aviv, Israel; ^2^Division of Rheumatology, Alan Edwards Pain Management Unit, McGill University Health Centre, Canada; ^3^Department of Medicine, H. Soroka Medical Center, 84101 Beer Sheva, Israel; ^4^Edwards Pain management Unit, McGill University Health Centre, Canada H3G 1A4; ^5^Department of Neurology, Universitätsklinikum Würzburg, 81865 Würzburg, Germany; ^6^Department Internal Medicine I, Klinikum Saarbrücken, 66119 Saarbrücken, Germany; ^7^Department of Psychosomatic Medicine and Psychotherapy, Technische Universität München, München, 90780 Würzburg, Germany

## Abstract

*Objective.* Current evidence indicates that there is no single ideal treatment for fibromyalgia syndrome (FMS). First choice treatment options remain debatable, especially concerning the importance of complementary and alternative medicine (CAM) treatments. *Methods.* Three evidence-based interdisciplinary guidelines on FMS in Canada, Germany, and Israel were compared for their first choice and CAM-recommendations. *Results.* All three guidelines emphasized a patient-tailored approach according to the key symptoms. Aerobic exercise, cognitive behavioral therapy, and multicomponent therapy were first choice treatments. The guidelines differed in the grade of recommendation for drug treatment. Anticonvulsants (gabapentin, pregabalin) and serotonin noradrenaline reuptake inhibitors (duloxetine, milnacipran) were strongly recommended by the Canadian and the Israeli guidelines. These drugs received only a weak recommendation by the German guideline. In consideration of CAM-treatments, acupuncture, hypnosis/guided imagery, and Tai Chi were recommended by the German and Israeli guidelines. The Canadian guidelines did not recommend any CAM therapy. *Discussion.* Recent evidence-based interdisciplinary guidelines concur on the importance of treatment tailored to the individual patient and further emphasize the need of self-management strategies (exercise, and psychological techniques).

## 1. Introduction

Treatment strategies for the management of fibromyalgia syndrome (FMS) include a variety of pharmacological and nonpharmacological therapies including complementary and alternative medicine (CAM) treatments [1, 2]. The extensive use of various therapies by most FMS-patients drives direct health care costs which are reported to be substantial in these patients [3, 4]. Evidence-based guidelines aim to guide health care providers and patients in the choice of treatment options. However, there remains a debate regarding the first choice of treatments for FMS. Previous evidence-based guidelines were formulated in 2005 by the American Pain Society (APS), in 2007 by the European League Against Rheumatism (EULAR), and in 2008 by the Association of the Scientific Medical Societies in Germany (AWMF). The EULAR guideline assigned the highest level of recommendation to a set of pharmacological treatments, whereas the APS and the AWMF guidelines assigned the highest level of recommendation to mostly nonpharmacological treatments which included aerobic exercise, cognitive-behavioral therapy, and multicomponent treatment, with amitriptyline identified as the only pharmacological agent strongly recommended [5].

The aim of the current review is to compare the recommendations of recent evidence-based interdisciplinary guidelines on the treatment of FMS with particular emphasis on the role of CAM-treatments as defined by the US National Institute of Health [6].

## 2. Material and Methods

### 2.1. Search of the Literature

A systematic search of the US Agency for Healthcare Research and Quality (AHRQ)'s (http://www.ahrq.gov/) American National Guideline Clearing House (NGC) (http://www.guideline.gov/), the Scottish Intercollegiate Guidelines Network (SGN) (http://www.sign.ac.uk/guidelines/index.html), and the Guidelines International Network (G-I-N) (http://www.g-i-n.net/) was conducted from January 2008 to February 2013, using the key words “Fibromyalgia” and “Fibromyalgia Syndrome.” Medline was also searched from January 2008 to February 2013 with the search terms “Guideline” [Publication Type] AND “Fibromyalgia” [Mesh]. A manual search of the guideline bibliographies and contacts to international FMS key opinion leaders was undertaken to verify that all published guidelines were identified.

### 2.2. Inclusion Criteria

To be included in our analysis, the guidelines had to meet the following criteria:

1. the guideline was commissioned by a scientific organization;
2. the guideline group was interdisciplinary and included at least the specialties rheumatology, pain medicine, and psychiatry or psychosomatic medicine or clinical psychology;
3. a systematic search strategy was outlined;
4. recognized criteria of classification evidence and recommendations were used;
5. the formal process for establishing recommendations (Delphi exercise, panel conference) was outlined;
6. the guideline commented on CAM-therapies as defined by the National Center of Complementary and Alternative Medicine [6].

### 2.3. Exclusion Criteria

Guidelines that included FMS with other diagnoses, such as chronic fatigue syndrome, myalgic encephalomyelitis, or somatoform disorders, were excluded.

### 2.4. Analysis of the Guidelines

Inclusion criteria and the composition of the steering committees and panels, search strategies, the classification of evidence and recommendations, the procedures for establishing recommendations, and the recommendations given by the guidelines that met inclusion criteria were assessed by two independent reviewers (MAF, WH). Levels of evidence were extracted according to the classification of the Oxford Center of Evidence-Based Medicine [7] (see Table 1). Grades of recommendation were classified according to the classification of the German National Disease Management Guidelines [8].

- Strong recommendation: the intervention should be offered to most of the patients.
- Recommendation: the intervention may be offered to the majority of patients. The intervention may not be offered to a substantial minority of the patients.
- Open recommendation: the intervention can be offered to a minority patients.

All discrepancies were rechecked and consensus achieved by discussion. If needed, a third reviewer was involved (JNA).

## 3. Results

### 3.1. Guideline Selection

The literature search yielded 24 citations (1 in NGC, none in SIGN, 2 in GIN, and 21 in Medline). FMS opinion leaders reported two guidelines. Three of these met our inclusion criteria: the 2012 Canadian Guidelines for the diagnosis and management of fibromyalgia syndrome [9], the guideline of the Association of the Scientific Medical Societies in Germany on the definition, pathophysiology, diagnosis, and treatment of fibromyalgia syndrome (AWMF) [10–15], and the Israeli guidelines for the diagnosis and treatment of the fibromyalgia syndrome [16]. The reasons for excluding other hits were as follows: duplications (*n* = 19); no criteria for establishing level of evidence outlined (*n* = 1); not commissioned by a scientific society (*n* = 1). 

### 3.2. General Principles of Treatment

All recommendations for general principles of treatment were based on (strong) expert consensus [EL 5, Grade D] if not otherwise indicated. All three guidelines agreed that treatment should be initiated with appropriate patient education on the nature of the symptoms and treatment options [9, 10, 16]. The Canadian and German guideline recommended that healthcare professionals should be empathetic, open, and honest and should not demonstrate negative attitudes. A therapeutic alliance would facilitate shared decision-making between the health care professional and the FMS-patient [9, 10] [EL3, Grade C].

All three guidelines recommended that attention should be paid to individual symptoms (e.g., pain, sleep problems, fatigue, and depression) in a patient tailored approach [9, 10, 16]. The German guideline emphasized consideration of patient comorbidities as well as patient preference in the selection of therapeutic measures [10]. All three guidelines recommended that patients should be encouraged to identify specific goals regarding health status and quality of life at the initiation of treatment with close monitoring and regular followup, particularly in the early stages of management [9, 10, 16]. The German and Canadian guidelines suggested that the overall clinical status (symptom reduction and functional improvement versus side effects and cost) should be regularly evaluated, with a proviso that therapy should only be continued in the setting of a positive benefit [10].

All three guidelines agreed that nonpharmacological strategies with active patient participation should be an integral component of the therapeutic plan [EL 1a, Grade A] [9, 10, 16]. The Canadian guideline stressed that persons with FMS should be encouraged to pursue as a normal life pattern as possible, using pacing and/or graded incremental activity to maintain or improve function, and those either working or with work potential should be encouraged to remain in the workforce [9].

### 3.3. Major Positive Recommendations (except CAM)

All three guidelines strongly recommended aerobic exercise, cognitive behavioral therapy, and multicomponent therapy (combination of exercise therapy with at least one psychological therapy) [9, 12, 13, 15, 16]. The German guideline gave a strong recommendation to low intensity strength training [13]. In consideration of pharmacologic treatments, anticonvulsants (gabapentin and pregabalin) and serotonin noradrenaline reuptake inhibitors were strongly recommended by the Canadian and Israeli guidelines, albeit that these drugs provide only modest relief of symptoms [9, 16]. In contrast, these drugs received only a weak recommendation by the German guideline [11] because these drugs have not been approved for the treatment of FMS in Europe (see Table 2).

### 3.4. Major Negative Recommendations (except CAM)

All three guidelines concurred that the use of strong opioids should be discouraged (EL 4, Grade A) [9, 11, 16]. The German guideline gave negative recommendations to antiviral agents (EL2b, Grade A), anxiolytics (EL2a, Grade A), cannabinoids (EL3a, Grade B), cold therapy (EL3b, Grade B), dopamine agonists (EL2a, Grade A), flupirtine (EL4, Grade B), hormones (e.g., corticosteroids, growth hormone, thyroid hormones) (EL3a, Grade A), hypnotics (EL3a, Grade A), interferon (EL2b, Grade A), ketamine (EL4a, Grade A), laser therapy (EL3a, Grade B), local anesthetics (EL3a, Grade A), milnacipran (EL1a, Grade B), monoaminooxidase inhibitor (EL1a, Grade B), muscle relaxants (EL1a, Grade B), nonsteroidal agents (EL1a, Grade B), neuroleptics (EL3a, Grade A), sodium oxybate (EL3, Grade A), transcranial electric stimulation (EL2, Grade B), transcutaneous electric stimulation (EL3, Grade B), and written emotional disclosure (EL2, Grade B) [12–14]. The Israeli guideline gave negative treatment recommendations for non-steroidal agents, systemic steroids, benzodiazepines, thyroid hormone replacement, and guaifenesin [16]. The Canadian guideline gave no specific negative treatment recommendations, but stated that evidence was lacking to support recommendation of many treatments which would constitute “off-label” use.

### 3.5. Recommended and Nonrecommended CAM-Therapies

Positive recommendations for CAM-therapies of the German and Israeli guidelines are outlined in Table 3.

The Canadian guideline gave a strong recommendation that patients should be informed that there is currently insufficient evidence to support the recommendation of complementary and alternative medicine (CAM) treatments as they have mostly not been adequately evaluated regarding benefit [9].

The German guideline gave negative recommendations to chirotherapy (EL3a, Grade B), dance therapy as monotherapy (EL2b, Grade B), homeopathy (EL1a, Grade A), magnetic field therapy (EL2a, Grade B), massage (EL2a, Grade B), mindfulness based stress reduction (EL1a, Grade A), nutritional supplement (algae and malic acid/magnesium preparations; anthocyanins; carnitine; *S*-adenosyl methionine, SAM; soya oil; vitamin-dietary mineral preparations) (EL3, GradeA), reiki (EL2b, Grade B), and relaxation training as single therapy (EL1a, Grade A) [13, 14]. There was a substantiated minority vote to recommend homeopathy (EL1a, Grade C) [4, 13].

The Israeli and Canadian guidelines gave no negative recommendation on distinct CAM-treatments [9, 16].

### 3.6. Graduated Approach

All recommendations for a stepwise treatment approach were based on (strong) expert consensus [EL 5, Grade D]. All three guidelines advocated a graduated treatment approach, however, in a different manner.

The Canadian guideline recommended that management of FMS should be centered in the primary care setting with knowledgeable healthcare professionals, and ideally, where possible, this care could be augmented by access to a multidisciplinary team or team member to provide support and reassurance. Specialist consultation, including referral to a sleep specialist or psychologist could be required for selected subjects, but continued care by a specialist was not recommended and could be reserved for those patients who had failed management in primary care or had more complex co morbidities [9].

The German guideline advocated a treatment approach adapted to the severity of FMS [10]. With reference to the German guideline on “nonspecific, functional, and somatic body complaints” [17], a distinction of mild, moderate, and severe forms of FMS, based on the number and intensity of somatic and psychological symptoms and the degree of disability, was made. Moderate and severe forms of FMS are due to somatic and mental comorbidities. The graduated treatment approach according to severity of FMS according to the German guideline [8] is as follows.

- Mild FMS: advice to continue increase physical, mental, and social activities (no specialist care, no recommendation for additional therapies).
- Moderate FMS: aerobic exercise; timely limited psychological (specialist care), and/or drug therapy.
- Severe FMS or moderate FMS without response to therapies mentioned above: day clinic or inpatient multicomponent therapy including psychotherapeutic and/or psychiatric therapy of mental comorbidities.
- Long-term management for all FMS: self-management without drugs.

Because the label “FMS” was rejected by the German Association of General and Family Medicine, the German guideline recommended that treatment and its coordination should, if possible, be performed by a doctor who has the necessary knowledge and experience in the treatment of FMS (e.g., pain medicine, psychosomatic medicine, and rheumatology). Treatment of FMS-patients should be usually on an outpatient basis. In the following situations, admission to a hospital was recommended.

- Inpatient treatment needs for comorbid physical and mental disorders.
- (Semi-)inpatient multimodal pain therapy.

Investigation of (semi-)inpatient rehabilitation measures was recommended, based on the criteria of the International Classification of Functioning (ICF), when

- participation in the labor force is at risk,
- participation in social life or ability for self-sufficiency is at risk,
- strongly recommended outpatient therapeutic measures are unavailable or insufficiently effective [10].

In the case of a response to drug therapy, after treatment duration of at least 6 months, a drug cessation trial should be considered for the patient. The patients who experience an improvement with aerobic endurance training should continue this training permanently [EL1a, Grade A] [10].

The Israeli guideline advocated a stepwise treatment approach depending on the response of the patient on first choice treatment options [16]. The Israeli guideline did not specify which specialty should treat FMS-patients.

Graded approach to treatment according to the Israeli guideline [14].

*Step  1*

- Education and explanation regarding the essence of the disorder and the principals of treatment.
- Instructions regarding graded aerobic exercise, adjusted to the functional level, and general health level of the patient.
- Referral to hydrotherapy/aquatic exercise.
- Start low dose amitriptyline 10–25 mg at bedtime.
- Refer for cognitive-behavioral treatment.

*Step  2*

Recommendations included in step 2 are based on a reassessment of the fibromyalgia patient's condition, about 12 weeks after initiating treatment.

- Treatment with a SNRI medication (duloxetine, milnacipran) *instead *of amitriptyline or addition of an SSRI medication (e.g., fluoxetine) *together with *treatment with Amitriptyline.
- Start treatment with pregabalin to improve sleep quality and reduce pain.
- Refer for balneotherapy.
- Add complimentary medicine modalities: Tai Chi and Yoga.

Consider combining more than one medication from different groups, as necessary. As needed, add analgesic medication—for example, tramadol.

## 4. Discussion

Recent guidelines for the treatment of FMS across three continents show remarkable similarities in the general principles of care and nonpharmacological first choice treatment options. Differences were mostly observed for recommendations on drug and CAM-treatment. A patient-tailored and stepwise treatment approach was the common focus of all three guidelines, with education of the patient and appropriate treatment goals forming the basis of any treatment. Within shared decision making, treatment choices should be driven by key symptoms, preferences and comorbidities of the patient, and potential side effects of a given treatment. Moreover, the availability and costs/reimbursement of treatments must be considered. The three guidelines agreed on the promotion of self-management strategies. Aerobic exercise, adapted to the general health status and functional level of the patient, and psychological techniques (e.g., relaxation and stress management) which can be acquired by participation in a cognitive-behavioral group therapy for chronic pain patients are the cornerstone of FMS-management with the highest level of evidence and strength of recommendation. Unrealistic (“cure”) and passive physical treatments (e.g., massage, “magic pill”) should be discouraged. The criteria for a stepwise treatment are severity of FMS (Germany), comorbidities of FMS (Canada), and response to previous therapies (Germany, Israel).

The differences regarding the strength of recommendation regarding drug therapy—despite evidence levels 1a for some anticonvulsants and some antidepressants—between Canada and Israel (strong recommendation) and Germany (open recommendation) can be explained by the different status of approval in the three countries, cultural differences regarding patient treatment expectations, and some differences in the criteria for recommendations. Even in the context of strong recommendation for these drugs in Canada and Israel, it is now recognized that drug treatment alone provides only limited benefit. Taking cost considerations and patient acceptance into account, amitriptyline, the only drug approved for the treatment of chronic pain in Germany, remains a reasonable treatment option in both Canada and Israel. The Canadian guidelines strongly recommended that all categories of antidepressant medications may be considered for use in FMS taking into consideration the individual characteristics of the patient, especially in the context of associated psychological distress. No drug has been approved for FMS-therapy in Germany. In contrast, duloxetine and pregabalin have been approved for FMS in Canada and Israel. These two drugs have been approved for some mental disorders in Germany. Besides, availability, efficacy, tolerability (drop out rates in studies), and safety were criteria to build recommendations in the German guideline. There is strong evidence for sustained but declining positive effects of aerobic exercise, CBT, and multicomponent therapy on some FMS symptoms [12, 13, 15]. The reduction of symptoms by drugs disappear in case of discontinuation [11]. In addition, drugs may induce side effects, often similar to symptoms of FMS, but at times more serious such as substantial weight gain or liver damage. It is for this reason that the German guideline favoured non pharmacological treatment and advocated timely limited drug therapy only in moderate and severe forms of FMS [10, 11].

Cannabinoid use in patients with chronic pain and FMS in particular remains controversial. With only the pharmacologic preparation nabilone having been tested in FMS, with some effect on sleep, but not consistent effects on global symptoms of FMS, the Canadian guides gave weak recommendation for a trial of pharmacologic cannabinoid, particularly in the setting of important sleep disturbance [9]. Herbal preparations of cannabinoids have not been formally tested in FMS and therefore received no recommendation from any of the guidelines. In contrast, the German guidelines recommended against the use of pharmacological cannabinoids by strong consensus citing the potential risks and lack of license in Germany as factors influencing this recommendation [11]. The Israeli guidelines state that current evidence is insufficient, make no treatment recommendations regarding cannabis and cannabinoid derivatives, and call for additional research into this issue [16].

Of note, all three guidelines discouraged the use of strong opioids [8, 10, 15].

CAM treatments are known to be used extensively by FMS patients [1, 2, 18], with differences between the recommendations of the three guidelines mostly related to the use of CAM. These differences may however not be as striking as first perceived due to categorizing of treatments differently between the three countries. Tai Chi was categorized as an exercise intervention by the Canadian guidelines, whereas it was evaluated as a CAM by the German and Israeli guidelines. Similarly, hypnosis and guided-imagery were evaluated as psychological interventions by the Canadians rather than as a CAM. Tai Chi, hypnosis/guided imagery and acupuncture, received a weak recommendation by the German and Israeli guidelines. In assessing Tai Chi as an exercise intervention, the Canadian guidelines acknowledged the value of the combined physical and mental component characteristics of Tai Chi, which is ideally suited to persons with FMS. Hypnosis with analgesia suggestion was evaluated as a psychological intervention in Canada with effect on pain, whereas guided imagery impacted self efficacy, but with a call for improved methodology of studies prior to a definitive statement regarding effect on key domains in FMS. The Canadian guidelines cited the immediate pain relieving effects of acupuncture, but without evidence for prolonged effect. But when combined with other treatments including exercise and tricyclic antidepressants (TCAs), acupuncture was associated with improvement in all measures of pain. 

Hydro- and balneotherapy was not recommended by the Canadian guideline but were recommended by the German and Israeli guidelines. Water therapy treatments are less familiar to the Canadian community, with less availability, whereas they have been in common use over centuries in Europe and the Levant. Attitudes of physicians towards these therapies might therefore be more positive in Germany and Israel where access to hot spring resorts is more available to the public. Studies have also shown short term benefits for some FMS symptoms, but with limitations of interpretation due to low quality of studies [13]. Overall, the use of most CAM-therapies (e.g., homeopathy, mindfulness-based stress reduction, nutritional supplements, and reiki) was discouraged by the German guideline.

## 5. Conclusions

It is reassuring that these three recent guidelines addressing treatments for FMS and developed independently across three continents show striking similarities. FMS cannot be cured by any therapy. There is consensus that self-management strategies will help patients to recognize and adapt to symptoms in order to preserve or improve their daily function and maintain quality of life. Although the large majority of CAM therapies are without evidence for effect, some such as Tai Chi, hypnosis/guided imagery, or meditative movement therapies may be incorporated into self-management strategies. In view of the long-term nature of FMS which requires treatment which can be safely prescribed over many years or decades, CAM appears to be an attractive adjunct in the therapeutic strategy of this challenging condition.

## Figures and Tables

**Table 1:** Levels of evidence according to the Oxford Center of Evidence-Based Medicine [7].

Level	Therapy/prevention, aetiology/harm
1a	SR (with homogeneity*) of RCTs
1b	Individual RCT (with narrow Confidence Interval)
1c	All or none^§^
2a	SR (with homogeneity*) of cohort studies
2b	Individual cohort study (including low quality RCT; e.g., <80% followup)
2c	“Outcomes” research; ecological studies
3a	SR (with homogeneity*) of case-control studies
3b	Individual case-control study
4	Case-series (and poor quality cohort and case-control studies)
5	Expert opinion without explicit critical appraisal, or based on physiology, bench research, or “first principles”

SR: Systematic review; RCT: Randomised controlled trial.

**Table 2:** Comparison of major positive treatment recommendations of the three guidelines.

	Canada		Germany		Israel	
	Level of evidence	Strength of recommendation	Level of evidence	Strength of recommendation	Level of evidence	Strength of recommendation
Aerobic exercise	Ia	A	Ia	A	Ia	A
Amitriptyline	Ia	A	Ia	B	Ia	A
Anticonvulsants (gabapentin, pregabalin)	Ia	A	Ia	C	Ia	A
Balneotherapy	No comment		Ia	B	Ia	C
Cognitive-behavioral therapy	Ia	A	Ia	A*	Ia	A
Multicomponent therapy	Ia	A	Ia	A	Ia	A
SNRI (duloxetine, milnacipran)	Ia	A	Ia	B/C**	Ia	A
SSRI	Ia	A	Ia	C	Neither positive nor negative recommendation	
Tramadol	IIa	C	No comment***		IIa	B

*If combined with exercise; **B in case of a comorbid depressive or generalized anxiety disorder; C in case without depressive or generalized anxiety disorder; ***In case of only one RCT with positive results, no recommendation was given.

SNRI: serotonin noradrenaline reuptake inhibitors; SSRI: serotonin reuptake inhibitors; TCA: tricyclic antidepressant.

A: strong recommendation: the intervention should be offered to most of the patients.

B: recommendation: the intervention may be offered to the majority of patients. the intervention may not be offered to a substantial minority of the patients.

C: open recommendation: the intervention can be offered to a minority patients.

**Table 3:** Positive treatment recommendations guidelines for complementary and alternative medicine treatments by the German and Israeli guidelines.

	Germany		Israel	
	Level of evidence	Strength of recommendation	Level of evidence	Strength of recommendation
Acupuncture	Ia	C	III	Not recommended
Biofeedback	IIa	C	No comment	
Hypnosis/guided imagery	IIIa	C	No comment	
Meditative movement therapies (Qi-Gong, Tai-Chi, Yoga)	Ia	A	III	C*
Relaxation training (combined with exercise)	Ia	A	No comment	

*Only Tai Chi recommended.

A: strong recommendation: the intervention should be offered to most of the patients.

B: recommendation: the intervention may be offered to the majority of patients. the intervention may not be offered to a substantial minority of the patients.

C: open recommendation: the intervention can be offered to a minority patients.
